# Characterization of Adhesives Bonding in Aircraft Structures

**DOI:** 10.3390/ma13214816

**Published:** 2020-10-28

**Authors:** Maria Grazia Romano, Michele Guida, Francesco Marulo, Michela Giugliano Auricchio, Salvatore Russo

**Affiliations:** 1 Department of Industrial Engineering, University of Naples Federico II, via Claudio 21, 80125 Napoli, Italy; mariagrazia.romano94@gmail.com (M.G.R.); marulo@unina.it (F.M.); 2 e-Xstream Engineering, Part of Hexagon’s Manufacturing Intelligence Division—Corso d’Italia 44, 00198 Rome, Italy; michela.giuglianoauricchio@hexagon.com; 3 Aircraft department, Leonardo SpA, 80038 Pomigliano d’Arco, Napoli; salvatore.russo@leonardocompany.com

**Keywords:** structural composite adhesive, lap shear test, cohesive zone modeling, multiscale model

## Abstract

Structural adhesives play an important role in aerospace manufacturing, since they provide fewer points of stress concentration compared to faster joints. The importance of adhesives in aerospace is increasing significantly because composites are being adopted to reduce weight and manufacturing costs. Furthermore, adhesive joints are also studied to determine the crashworthiness of airframe structure, where the main task for the adhesive is not to dissipate the impact energy, but to keep joint integrity so that the impact energy can be consumed by plastic work. Starting from an extensive campaign of experimental tests, a finite element model and a methodology are implemented to develop an accurate adhesive model in a single lap shear configuration. A single lap joint finite element model is built by MSC Apex, defining two specimens of composite material connected to each other by means of an adhesive; by the Digimat multi-scale modeling solution, the composite material is treated; and finally, by MSC’s Marc, the adhesive material is characterized as a cohesive applying the Cohesive Zone Modeling theory. The objective was to determine an appropriate methodology to predict interlaminar crack growth in composite laminates, defining the mixed mode traction separation law variability in function of the cohesive energy (G_c_), the ratio between the shear strength τ and the tensile strength σ ($\beta_{1}$), and the critical opening displacement υ_c_.

## 1. Introduction

Adhesive bonding is one alternative to traditional methods with advantages in areas such as mitigation of galvanic corrosion, reducing stress concentrations, and enhanced fatigue strength and it has been a consolidated standard in the automotive, railway, aerospace, and many other industries for several years. Thanks to innovative adhesives, in industrial production today, it is possible to glue the most disparate materials, such as metals, plastics, or glass. However, conventional joining methods such as welding and mechanical fasteners are often unsuitable for use with dissimilar materials; moreover, in most of these production processes, the usage of adhesive bonding enables a reduction in the weight of the final products. Generally, adhesives for aerospace use are typically applied in the form of either a paste or a film. Pastes with high viscosities tend to form thicker bond lines and, therefore, fill and bridge gaps between bonded surfaces and can offer significantly greater elongation and impact resistance [1,2].

Considering that the traditional joining methods can only operate on materials above certain minimum thickness values to prevent damage (e.g., tearing, bending, burning) in the material during joining [3], adhesives are advantageous as they can be applied to materials of any thickness, reducing the presence of the holes, which results in stress concentrations; these aspects make bonding a very convenient tool to connect the parts together, but bonding can lead to substantial errors in the assessment of rigidity and stress state of the assembly [4]. In fact, the bonded joints are also affected by numerous disadvantages such as, for example, structural sensitivity to peeling and tearing loads with respect to tensile and shear loads; the need for careful preparation of the surfaces of the adherents, a very long curing time, direct inspection not being applicable, and non-destructive tests having to be carried out; the repair of faulty joints is practically impossible; the maximum operating temperature of a bonding can be very limited; the durability of a bonded joint is very dependent on environmental conditions (temperature values and humidity concentrations); the adhesives can be sensitive to the chemical attack of some solvents.

Different modeling of adhesively bonded joints has been approached over the years, aiming for a more accurate description and simpler calibration processes.

One approach consists of discretizing and modeling the adhesive line with a mesoscopic material model, characterized by a constitutive law describing the relation between stresses and strains in the material. This method based on the fracture mechanism is resulted to be incorrect in the case of non-linearity [5]. Cohesive Zone Modeling (CZM) solves these limits, simulating and analyzing damage initiation and propagation in bonded joints and laminates [6]. The elastic–plastic fracture mechanics can be evaluated using the J-integral method or the Virtual Crack Closure Technique (VCCT) in finite element codes [7].

The CZM technique was introduced by Dugdale and Barenblatt [8]. In their formulation, it is considered the presence of a plastic area ahead of the crack tip with constant stress equal to yielding strength, and as the cohesive forces separate, which occur when material elements are being pulled apart, the traction first increases until a maximum is reached and then, reduces to zero, which results in complete separation. Then, Hillerborg introduced a modified model assuming that the stresses vary during the deformation process and predicting crack initiation, which takes place when the tensile stress reaches the tensile strength [9]. The CZM approach evaluates the elastic loading, initiation of damage, and further propagation due to local failure within a material. This model is based on a relationship between stresses and relative displacements (in tension or shear) connecting paired nodes of the cohesive elements, to simulate the elastic behavior up to the tension or shear strength and subsequent softening, to account for the gradual degradation of material properties up to complete failure. By varying the cohesive energy and considering non-linear effects [10,11], with the elastic orthotropic properties of the materials, the numerical analysis completely simulates the adherends and the triangular cohesive model for the adhesive. Therefore, it improves the level of accuracy of the technique for the strength prediction of bonded joints, helps the understanding of how to surpass the limitation of stress/strain and fracture criteria, and finally, simulates the damage growth.

The behavior and the performance of the bonded joints are influenced and controlled by several factors, such as: the configuration of the joint, the physical and mechanical properties of the materials, the compatibility between the constituents, the loading particularities, and the environmental exposure conditions [12]. The structural response of these types of adhesive joints generally refers to the relation between the stresses and strains that develop along the bond length [13]. The distribution of these two components can be determined by applying either analytical or numerical approaches, each of them having their own advantages and drawbacks [14].

Several approaches in the literature allow the prediction of the strength of adhesively bonded joints and the relative failure criterion [15]. Since 1938, Gustafson [16] presented a model for adhesive bonded joints where the adherend bending and shear deformations were not considered. These limitations were implemented by Goland and Reissner [17], considering the importance of the adherend bending favoring the peeling in the adhesive layer, in addition to the shear stress [15]. Then, the Hart-Smith [18] model considered the adhesive elastoplastic behavior concerning the adhesive layer, introducing also thermal effects. Renton and Vinson [19] included first-order shear deformation in their analysis without evaluation of the coupling of the shear and bending responses of the joint, which is very important for the stress distribution evaluation. Delale [20] was the first to evaluate the importance of the coupling between bending and extension, while Allman [21] implemented the adhesive stress-free boundary condition in order to realize the full equilibrium equations for the adherends. Adams and Mallick [22] used Allman’s method to expand a one-dimensional finite element solution considering non-linear adhesive behavior.

Yang and Pang [23] also produced analytical models by using classical laminated plate theory with first-order shear deformation to evaluate asymmetric and symmetric single lap joints during tensile and bending loading. Wu [24] investigated the behavior of joints linked with different adherends, evaluating the variability of the thickness and dimension, defining more appropriate differential equations.

Mortensen and Thomsen [25] modeled the adherends as beams, the composite adherends as orthotropic laminates, and the adhesive as a linear elastic material to make applicability to the composite joints uniform. More recently, Zhang [26] introduced a methodology for multi-axial stress analysis of composite joints, referring to the approach of Mortensen and Thomsen [27] that computed the in-plane and interlaminar stresses in the adherends.

In this work, starting from the reference standard, a single lap joint finite element model was built; this consists of two specimens of composite material connected to each other by means of an adhesive. The composite material was characterized in Digimat; the finite element model, the geometry, and the mesh were built in MSC Apex; and finally, the adhesive material was characterized as a cohesive in MSC’s Marc using the Mentat preprocessor according to the Cohesive Zone Modeling theory, validated by comparing results with standardized thin coupon tests prior to further simulation.

A suitable data reduction scheme was developed to measure the adhesive joints’ strain energy release rate under mixed pure mode I and II loading. The results are discussed in terms of their relationship with adhesively bonded joints and thus, can be used to develop appropriate approaches aimed at using adhesive bonding and extending the lives of adhesively bonded repairs for aerospace structures.

## 2. Failure Analysis

Each method predicts the failure strength and modes in different ways, complicating the process of correlating the results with each other. The finite element methods analyze the failure in four different approaches: fracture mechanics, continuum mechanics, damage mechanics, and an extended finite element method [28]. However, these models based on progressive damage are able to simulate the joint behavior with a certain reliability [29].

### 2.1. Continuum Mechanics

The continuum mechanics investigate the material failure analysis, correlating the maximum values of stress, strain, or strain energy resulting from finite element analyses to the critical values of the material indicating the material failure.

The criteria about the continuum approach are limited to continuous structures; therefore, they have difficulty in the presence of defects or cracks which are visible in bonded joints, defined like singular points. The continuum approach is adequate for continuous structures, it is limited in the presence of defects or discontinuities that, in the modeling, can generate singularity points [6].

Adams and Harris [30] studied the singularity by intervening on the stress peak (or plastic strain energy density for the non-linear analyses). In this way, the problem shifts towards deciding how much to approximate the stress levels to avoid affecting the strength of the joint. From the moment that the mechanics of the continuum are applied at a certain distance, the knowledge of the exact form applied far away from the singularity becomes fundamental. This method is known as distance stress or strain [31].

### 2.2. Fracture Mechanics

Toughness is considered as failure criterion; therefore, it is possible to determine the values of toughness function to predict the crack path and to estimate the strength of the joint for different loading conditions [32].

The fracture mechanics provides two distinct ways to analyze the way of breaking of materials and the way of propagation of the fracture. LEFM is dedicated to linear elastic fractures, such as Griffith’s criterion [33], so the non-linearity is neglected and the failure assumes a linear elastic behavior up to the point of failure and simple failure criteria are used to indicate crack growth once reached [6]. Contrarily, in the presence of ductile materials, the Elasto-Plastic Fracture Mechanics (EPFM) method is more suitable is more suitable, in which there is a material subject to plastic deformation before failure; in this specific case, the displacement of the opening displacement is an important parameter to evaluate, such as the spherical zone or the plastic radial [28] of the crack tip and a radial area or spherical plastic that was incorporated the slot. Among the most studied and continuously improved methods are J-integral and Virtual Crack Closure, in which the problem is due to the singularity of the deformation [2] and the hypothesis that the stress is zero at the boundaries in proximity of the plastic zone [8].

### 2.3. Extended Finite Element Method

The extended finite element methods (XFEM) have been adapted to model crack propagation since 1999 [34]; over the years, this approach has allowed the extension of applications also in the simulation of 3D damage [35], improving the function of the basis for incorporating singularities in EPFM, and studying crack propagation and frictional contact modeling incorporating geometric non-linearities and applications, [36].

### 2.4. Damage Modeling

The damage modeling methods do not require post processing iteration loops to achieve a complete response result of the collapsed structure. In general, the cohesive zone is evaluated and the approaches can be different depending on whether the damage is being evaluated in a finite region (continuous approach) or locally by limiting the damage to a volume or to a surface equal to zero (local approach), as described by the theories of Dugdale, Barenblatt, and Hillerborg [8,9], where the cohesive law takes into account the area of the tensile–separation curve (toughness) and the cohesive force or the displacement that measures the fracture deformation of the cohesive zone.

Sørensen and Jacobsen [37] determined the cohesive laws of a sample subjected to a double cantilever beam (DCB) test by measuring the displacement with strain gauges, differentiating the integral J (determined by the load) with respect to the displacement in the end opening.

Li [38] instead introduced a cohesive law to model the fracture in mode I and obtained very encouraging results with numerical simulations. The CZM has become a privileged method for predicting static, fatigue, and damage propagation and the advantages of this approach are numerous also because it allows the study of alternative mixed adhesive joints.

## 3. The Single Lap Shear Test

The experimental tests were performed on different sets of adhesively bonded, carbon unidirectional fibers lap-shear coupon specimens that are fabricated for ultimate strength testing according to the ASTM D3165 standards [39]. The goal of the single lap shear test is to test the properties of the adhesive when subjected to tensile load. The specimens are provided with tabs, according to the ASTM reference, to avoid the misalignment of the load.

Before the test, there was a procedure to be followed to prepare the specimens and the bonding. First of all, before joining the elements with the adhesive, the composite specimens must be cut, cleaned, and cured before joining the elements with the adhesives. After that, the adhesive was applied and, with pressure, the whole system was closed.

**Figure 1:**
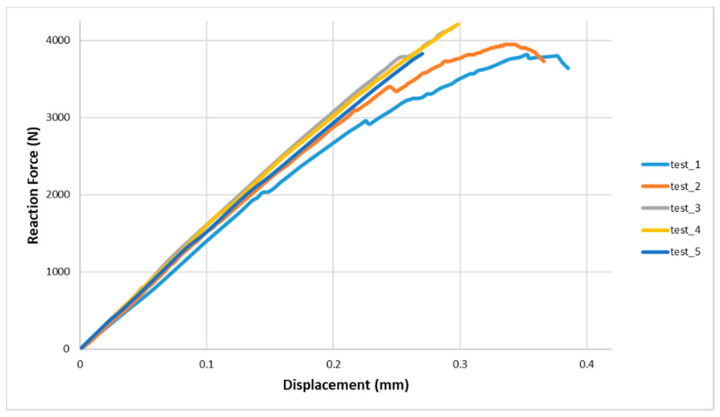
Experimental results from the single lap shear test.

All of the tests were performed on electromechanical loading frame at room ambient temperature, and finally, the static tensile load was applied. Five tests were carried out under the same conditions for different but identical samples to guarantee the reliability of the results; as reported in the following Figure 1, the test was preceded until a maximum load was achieved and followed by a sudden load drop, indicating specimen failure characterized by a mix of adhesive and cohesive failures. The adhesive was shown to be very ductile (more than 20% shear strain to failure), so yielding and sustaining further load until eventually its shear strain failed was attained.

The results indicated that the epoxy resin-based hybrid polymer composites, used like adhesive bonding for the samples, showed overall good behavior in terms of both mechanical strength and adhesive stiffness (energy and maximum displacement). Cracking gradually appeared during the experimental tests, as determined from the cracking sounds emitted. A sample presented more scatter than the other coupons because it was more sensitive to defects (defect of manufacturing) or characterized by not perfect clamping. However, the failure surface did not contain any noticeable void.

## 4. Numerical Simulations

**Figure 2:**
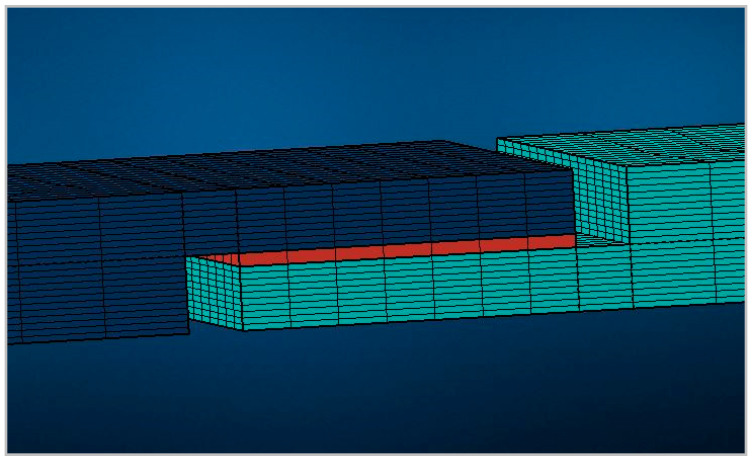
Single lap joint model and mesh.

The numerical simulations have been conducted by FEM analysis (Marc 2017.1.0, MSC Software Company, Newport Beach, California), ref. [40], considering two composite coupons joined by an adhesive component, as reported in Figure 2.

The experimental tests made it possible to define the material properties, obtaining the calibration of the material card in terms of the fiber and matrix properties. The accurate description of the local behavior of the Continuous Fiber material was investigated using Digimat (2018.1, e-Xstream engineering, Hautcharage, Luxembourg) and Marc (2017.1.0, MSC Software Company, Newport Beach, CA, USA) was used to characterize the property of the cohesive component. The finite element model and the mesh of the single lap joint were built in MSC Apex (Harris Hawk, MSC Software Company, Newport Beach, CA, USA). The mesh was coarse in the edge and was finer in the joint because it was the zone subjected to the analyses. The finite element model consisted of hex solid elements, and the adhesive material was modeled as a cohesive. The two coupons and the adhesive were joined together with a cohesive contact of glued type, and in order to replicate the test procedure, the boundary conditions were considered fixed on one edge, while the other was loaded by an axial displacement. The analysis was a non-linear structural static with a constant time step.

**Figure 3:**
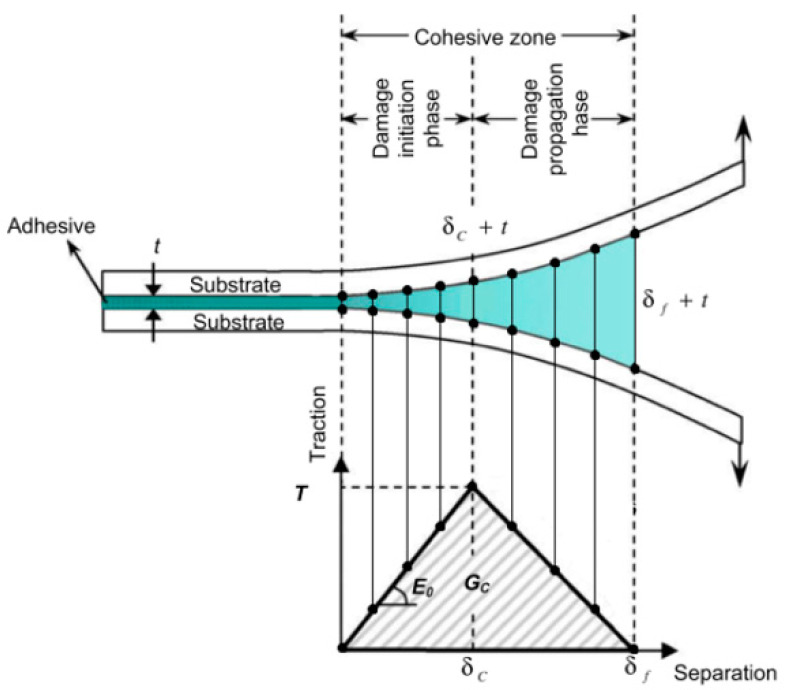
Traction law in function of the displacement for an adhesive joint.

The theory used for the bonding analysis was the Cohesive Zone Modeling (CZM), which relates traction to separation at an interface where a crack initiates. According to this theory, a cohesive element model can be defined with different laws that describe the relationship between the displacement δ and the traction law T. Crack initiation is related to the cohesive energy. When the area under the traction–separation curve reaches fracture toughness, the traction tends to zero and new crack surfaces result. Figure 3 defines a mixed mode triangular traction–separation response, and the area below the curve is the cohesive energy G_c_, while subscript c and f concerned critical and failure parameters and the values are related to i = I, II, and III for the pure modes of fracture.

The maximum traction load is a function of the cohesive energy and of the critical opening displacement *υ_c_*. In order to consider a different behavior of the material in the directions of tension and shear, a parameter $\beta_{1}$ was introduced, which is the ratio between the maximum shear stress τ and the maximum stress σ. In the same way, a different value of the shear and tension cohesive energy by introducing a parameter $\beta_{2}$ was considered, which is the ratio between the cohesive energy in the shear direction GII and the cohesive energy in the normal direction to the plane *G_I_*.

The subscripts I and II refer to two of the three ways in which the fracture can occur: mode I indicates a tension fracture; mode II shear; mode III, tear. Mixed modes that are combinations of the three main ones are also possible.

## 5. Methodology

**Figure 4:**
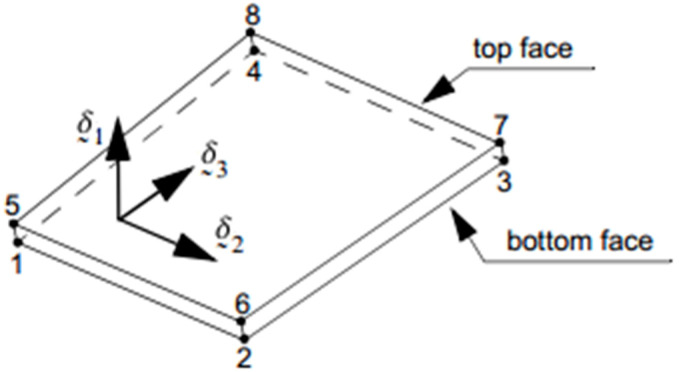
3-D Linear Interface Element.

For the CZM technique, the tractions are a function of the relative displacements of the upper and lower edges, as seen in Figure 4; therefore, the relative displacements are in the normal direction and in the two cutting directions. Each relative displacement is then expressed in the distance between the centers of the faces or ends of the top and bottom element.

$$\{\begin{array}{c} \begin{array}{c} \nu_{n}=u_{1}^{top}-u_{1}^{bottom}\\ \nu_{s}=u_{2}^{top}-u_{2}^{bottom}\\ \nu_{t}=u_{3}^{top}-u_{3}^{bottom} \end{array}\\ \nu =\sqrt{\nu_{n}^{2}+\nu_{s}^{2}+\nu_{t}^{2}} \end{array}$$

where displacements *u* are relative in normal $\nu_{n}$, shear $\nu_{s}$, and tear $\nu_{t}$ directions.

**Figure 5:**
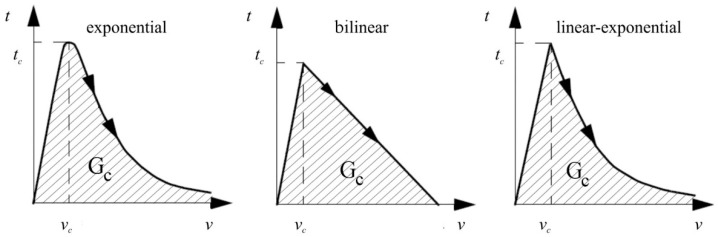
Types of cohesive models.

The traction *t* on opening displacement *v* is a function of exponential, bilinear, and linear–exponential (Figure 5), and the area below the functions represents the critical strain energy release rate *G_c_*.

The traction is a function of the effective relative displacement, and it presents a reversible behavior before and an irreversible behavior after the critical opening displacement $\nu_{c}$.

The irreversible behavior is characterized by an increasing damage law value from 0, being damage initiation, to 1, being damage propagation. The Marc code considers elastically damaged reloading behavior and it uses a newly calculated stiffness from the origin to the last point of the element on the traction law.

Considering that the traction laws are characterized by a bilinear, it is:

$$T=\{\begin{array}{cc} \frac{2G_{c}}{\upsilon_{m}}\frac{\upsilon }{\upsilon_{c}}, & \;\;0\leq \upsilon \leq \upsilon_{c}\\ \frac{2qG_{c}}{\upsilon_{m}}\frac{\upsilon_{m}-\upsilon }{\upsilon_{m}-\upsilon_{c}}, & \;\;\nu_{c}<\upsilon \leq \upsilon_{m}\\ 0, & \;\;\upsilon >\upsilon_{m} \end{array}$$

where traction is *T*, maximum effective opening displacement $\upsilon_{m}$ for the bilinear model and exponential decay factor q for the linear–exponential model, critical energy release rate *G_c_*, effective relative displacement υ, and critical opening displacement $\;\upsilon_{c}$.

The calibration of the adhesive correlating the experimental curves obtained by the single lap shear test is the objective, and in particular, from the five experimental curves given, it has obtained the interpolating second order curve; it was assumed as the reference curve for the numerical simulation.

The involved parameters to the adhesive calibration are reported in the following Table 1:

To start the adhesive calibration, three different cases with different assumption are followed, as reported in Table 2.

## 6. Cohesive Energy

The Cohesive Energy, $G_{c}$, is the energy release rate and it is defined as the area below the stress–displacement curve. By varying the cohesive energy, this factor influences the curve slope: increasing $G_{c}$, the slope increases; decreasing $G_{c}\;$, the curve slope decreases.

### 6.1. Critical Opening Displacement

The Critical Opening Displacement, $\upsilon_{c}$, is the critical displacement at which the maximum load corresponds. The $\upsilon_{c}\;$ is an unknown parameter and it is manually calibrated by varying the other parameters to obtain numerical curves with the same slope or the same maximum load of the experimental reference curve.

By varying the Critical Opening Displacement, this one is the parameter that better influences the maximum load value: by increasing $\upsilon_{c}$, the maximum load decreases; by decreasing $\upsilon_{c}$, the maximum load increases.

### 6.2. Shear to Normal Stress Ratio

*The Shear to Normal Stress Ratio,*$\;\beta_{1}$, is the ratio between the shear strength τ and the tensile strength σ.

### 6.3. Shear to Normal Energy Ratio

The Shear to Normal Energy Ratio, $\beta_{2}$, is the ratio between the cohesive energy in the shear and normal direction $G_{II}$ and $G_{I}$.

By varying the Shear to Normal Energy Ratio value, this is the parameter that better influences the displacement value corresponding to the maximum load: increasing $\beta_{2}$, the displacement value corresponding to the maximum load increases; decreasing $\beta_{2}$, the displacement value corresponding to the maximum load decreases.

#### 6.3.1. Calibration: Case (a)

Referring to the previous tables, in the first case, $\upsilon_{c}$ is the only unknown parameter. It will be varied in order to obtain the numerical curves with the same slope or the same maximum load of the reference curve.

**Figure 6:**
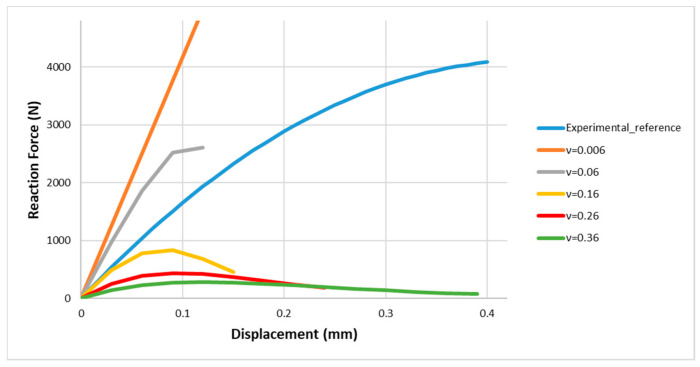
Numerical curves of the force in function of the displacement, by varying the critical opening displacement, assumed $G_{c}=\;G_{I}=G_{II}$ and $\beta_{2}=1$.

The maximum traction decreases when the critical opening displacement increases, considering a larger distance between nodes for a CZE to initiate damage, as reported in Figure 6. By varying the Critical Opening Displacement (CTOD), it is clear that the numerical curves obtained are far from the experimental reference curve; therefore, the calibration type (a) is not considered and an alternative calibration is to be considered.

#### 6.3.2. Calibration: Case (b)

In this case, two unknown parameters are considered: $\upsilon_{c}$ and $\beta_{2}$.

The procedure followed is:

1. Assign a $\beta_{2}$ value;
2. Obtain $G_{II}$ from the $\beta_{2}$ assigned, where the cohesive energy is considered ($G_{I}=G_{c})$;
3. Calibrate $\upsilon_{c}$ to obtain a numerical curve with the same slope or the same maximum load of the reference curve;
4. Assign a new value about $\beta_{2}$ and the procedure is repeated until from point 3, one is obtained with the same slope and the same maximum load of experimental reference.

**Figure 7:**
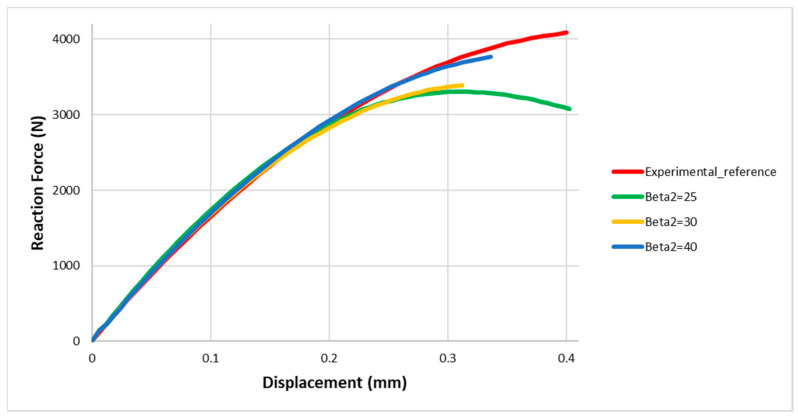
Numerical curves of the force in function of the displacement, by varying $\beta_{2}$, assumed $G_{c}=G_{I}$.

It is important to note that it is not possible in the Figure 7, or physically realistic, to have the cohesive element regard just pure Mode I or pure Mode II. Therefore, the element characteristics always calculate the mixed mode crack propagation using the ratio between the cohesive energy release in normal (Mode I) and shear direction (Mode II).

From this type of calibration, the numerical curves are close to the reference curve only when very high values (up to 30) are assigned to $\beta_{2}$: this means that the cohesive energy in the shear direction is at least 30 times higher than the cohesive energy in the tensile direction and this is physically impossible; therefore, this type of calibration is excluded.

#### 6.3.3. Calibration: Case (c)

In the last case, the independent variables are $\upsilon_{c}$ and $\beta_{2}$; $G_{c}$ is a variable dependent on $\beta_{2}$.

The procedure to be followed is the same as the (b) case, with the difference that, in this case, the value obtained of $G_{II}$ from point 2 coincides with the cohesive energy in order to calibrate $\upsilon_{c}$ and to obtain a numerical curve with the same slope or the same maximum load of the reference curve.

**Figure 8:**
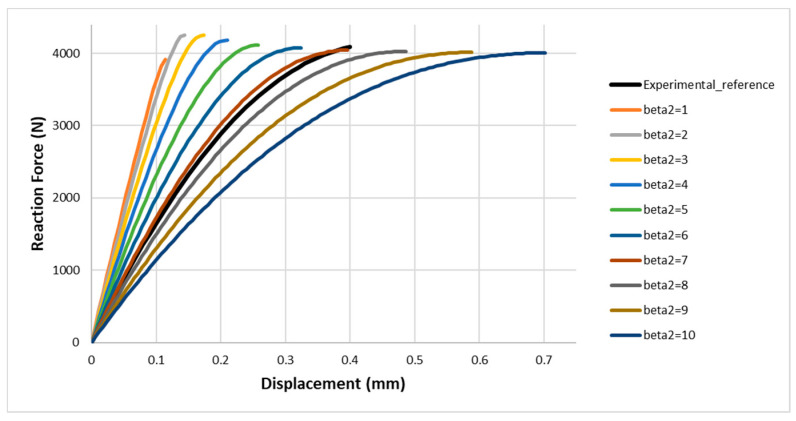
Numerical curves of the force in function of the displacement, by varying $\beta_{2}$ in the range [1; 10], assumed $G_{c}=G_{II}$.

Assigning $\beta_{2}$ value in the range from 1 to 10 (physically possible), the reference curve is between the numerical curves obtained for $\beta_{2}\in \left[6;8\right]$, as reported in Figure 8.

**Figure 9:**
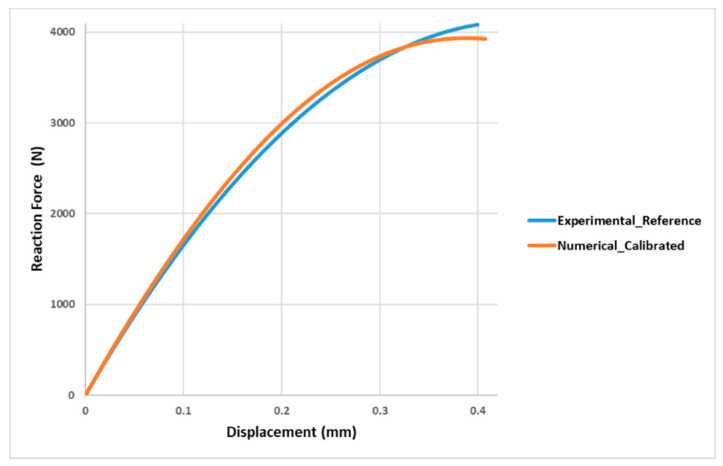
Experimental reference curve and numerical calibrated curve of the force in function of the displacement, assumed $G_{c}=G_{II}$.

Following the calibration described above, $\beta_{2}$ is varied in the new range, by restricting it gradually, Figure 9. In conclusion, the adhesive is calibrated for:

- $\beta_{2}$ = 6.8;
- $\upsilon_{c}=0.14.$

In practice, a value of *β*_2_ ≥ 1 is most common, because in mode II, more work of separation is measured due to additional dissipation mechanisms at the interface. The value of the Critical Opening Displacements $\upsilon_{c}$ is related to both I and II modes and this parameter is fit using the numerical simulations.

## 7. Conclusions

In this work, the attention is focused on the definition of a methodology to predict interlaminar crack growth in composite laminates in order to simulate the capability of the onset and the propagation of delamination. A finite element model of two specimens in composite material joined by an adhesive is presented, comparing the results by lap shear tests in order to virtually characterize the adhesive. The single lap joint was used as a kind of joint due to its simple geometry; considering the single lap joint as an assembly made of three parts, two adherends, and an adhesive, it was possible to reason about the effect on the strength of the adhesive joint. This analysis was numerically performed by MSC Marc, which was validated through the experimental tests. The adhesive material was characterized as according to Cohesive Zone Modeling, defining the linear traction–separation law varying the cohesive energy (*G_c_*), the ratio between the shear strength τ and the tensile strength σ ($\beta_{1}$), and the critical opening displacement *υ_c_*. Only one of these gave physically plausible results in comparison with the experimental reference and the parameters of the adhesives were determined for modeling of bonded joints using the cohesive crack model.

With a fixed cohesive energy, the critical opening displacement was evaluated to guarantee numerical–experimental comparison. Increasing the critical energy release rate between failure mode I and mode 2 has a larger influence than the increase in opening displacement and defining the adhesive’s influence in damage onset and growth. Considering that a larger critical energy release rate requires more area to cover and to fully damage a CZE, which is also related to the opening displacement between modes, this justifies because a larger gap is necessary to fully damage a CZE.

To this end, this research will be applied to develop a scripting procedure through which the adhesive calibration process can be automated.

## Figures and Tables

**Table 1:** Parameters involved in the characterization.

Parameters	Known	Unknown (To Calibrate)
Cohesive Energy $G_{c}$		x
Critical Opening Displacement $\upsilon_{c}$		x
Shear to Normal Stress Ratio $\beta_{1}$(τ/σ)	x	
Shear to Normal Energy Ratio $\beta_{2}$ ($G_{II}/G_{I})$		x
Cohesive Energy in the normal direction $G_{I}$	x	
Cohesive Energy in the shear direction $G_{II}$		x

**Table 2:** Assumption (a), (b), (c) for the adhesive characterization.

Case	Assumption	To Calibrate
a	$G_{c}=G_{I}=G_{II}$ $\beta_{2}=1$	$\upsilon_{c}$
b	$G_{c}=G_{I}\neq G_{II}$ $\beta_{2}=G_{II}/G_{I}$	$\upsilon_{c}$; $\beta_{2}$
c	$G_{c}=G_{II}\neq G_{I}$ $\beta_{2}=G_{II}/G_{I}$	$\upsilon_{c}$; $\beta_{2}$
